# Inhibitory Effects of* Prunella vulgaris* L. Extract on 11*β*-HSD1 in Human Skin Cells

**DOI:** 10.1155/2018/1762478

**Published:** 2018-10-04

**Authors:** Kyung-Baeg Roh, Deokhoon Park, Eunsun Jung

**Affiliations:** Biospectrum Life Science Institute, Yongin 16827, Republic of Korea

## Abstract

Glucocorticoids are a risk factor for age-induced skin structure and function defects, and the glucocorticoid-activating enzyme, 11*β*-hydroxysteroid dehydrogenase 1 (11*β*-HSD1), represents a promising therapeutic target.* Prunella vulgaris* L. (PV) is a perennial and an edible herbaceous plant normally cultivated in Asia and Europe. A recent study demonstrated a broad range of biological activities of PV including immune modulatory, antiviral, antiallergic, anti-inflammatory, antioxidant, and antidiabetic. However, little is known about the inhibitory effect of PV on 11*β*-HSD1. In this study, we investigated the inhibitory effect of* Prunella vulgaris* L. extract (PVE) and the underlying mechanism of 11*β*-HSD11 inhibition. Consistent with these results, cortisol levels were also reduced by PVE* in vitro*. The cortisone-induced translocation of glucocorticoids receptor (GR) was also attenuated. In addition, PVE inhibited a cortisone-mediated decrease in collagen content in skin. Collectively, these results suggest the beneficial effects of PVE in maintaining skin integrity.

## 1. Introduction

Glucocorticoid (GC) hormones are released in response to various physiological stressors and psychological stress [1]. It regulates various biological processes which induce diverse responses including differentiation, proliferation, and apoptosis [2]. The major GC, hydrocortisone, or cortisol is mainly synthesized and secreted in the adrenal cortex and regulated by adrenocorticotropic hormone (ACTH) under the control of hypothalamic-pituitary-adrenal (HPA) axis [3]. Various stressors, such as inflammation, viral infection, or feeling of strain and pressure, can stimulate the HPA axis [4]. Extra-adrenal production of cortisol has recently reported in various tissues [5]. Skin cells have also been reported to synthesize cortisol through activation of the enzyme, 11*β*-HSD1 [6, 7].

In skin, excessive GC production leads to marked skin integrity, including thinning and the dermal-epidermal junction (DEJ) flattening, decreased dermal cellularity, reduced collagen content, and dermal fibroblast proliferation [8–11]. Cortisol levels are regulated by isoenzymes of 11*β*-hydroxysteroid dehydrogenase 1 (11*β*-HSD1), which catalyzes the intracellular conversion of inactive cortisone into active cortisol [12].

Blockade of 11*β*-HSD1 resulted in a significant improvement in age-induced skin structure and function defects, accompanied by accelerated wound healing [7, 13]. These studies suggest that inhibition or downregulation of 11*β*-HSD1 provides beneficial effects for maintaining skin integrity.

*Prunella vulgaris* L. (PV) is a perennial and an edible herbaceous plant normally cultivated in Asia and Europe. PV has been used as a folk remedy for alleviating sore throats, reducing fever, and promoting wound healing [14]. In Asian countries, it has been used as a food or tea for a long time. A recent study showed that PV has a broad range of biological activities including immune modulatory, antiviral, antiallergic, anti-inflammatory, antioxidant, and anticancer [15–20]. PV has also been reported to be useful in the treatment of diabetes [21, 22]. However, little is known about the inhibitory effect of PV on 11*β*-HSD1. In this study, we investigated the inhibitory effect of PV on 11*β*-HSD11 and its underlying mechanism.

## 2. Materials and Methods

### 2.1. Preparation of* Prunella vulgaris* L. Extract

*Prunella vulgaris* L. (PV) was harvested in Jeju Island, Korea, from June to July and authenticated by Dr. Yong-Hwan Jung, Jeju Biodiversity Research Institute, Jeju Techno Park, Korea, where a voucher specimen (Voucher No. JBRI 150924-01). The aerial part of the plant was dried and grounded to a fine powder. Water extract of PV (10% w/v) was prepared by overnight extraction in sterile water at 40 ~ 50°C under the condition of slow shaking. The extracts were filtered through filter paper and then frozen on a freezing tray for 48 h. A perfectly dried extract of PV was obtained after freeze drying of the liquid extract for 60 h. The obtained extract was dissolved in distilled water for further experiments [23].

### 2.2. Cell Culture and Reagents

Human keratinocytes, HaCaT and normal human fibroblasts, and NHFs were maintained in DMEM (HyClone, Logan, UT, USA), containing 10% FBS and 1% penicillin/streptomycin at 37°C, under 5% CO_2_. Human epidermal keratinocytes (HEKn) were maintained in EpiLife (Invitrogen, Carlsbad, CA, USA), containing Human Keratinocyte Growth Supplement (HKGS, Invitrogen, USA) at 37°C, under 5% CO_2_. Cortisone and BVT.2733 were obtained from Sigma Aldrich (St. Louis, MO, USA). 11*β*-HSD1 promoter reporter clone was purchased from GeneCopeia (Rockville, MD, USA). Anti-GR/NR3C1-PE was purchased from Novus Biologicals (Littleton, CO, USA).

### 2.3. High-Performance Liquid Chromatography

The aqueous extract of PV was quantitatively analyzed by HPLC. The HPLC system (Shimadzu, Kyoto, Japan) with a CBM-20A controller, LC-20AD pump, SPD-M20A PDA detector, and a SIL-20A autosampler was used for the analysis of PV. Data collection was performed using Shimadzu Lab Solution. All chromatographic separations were performed on a CAPCELL PAK C_18_ UG120 column (250 mm × 4.6 mm, 5 *μ*m, Shiseido, Tokyo, Japan) with 0.1% acetic acid/acetonitrile gradient system at ambient temperature with detection at 254 nm. The amount of rosmarinic acid and caffeic acid in the water extract of PV were determined.

### 2.4. Cell Viability Assay

Cell viability was measured using the MTT (3-[4,5-dimethylthiazol-2-yl]-2,5-diphenyltetrazolium bromide, USB Corp., Cleveland, OH, USA) assay. Cells were plated in triplicate wells of 24-well plates and incubated overnight. Subsequently, the cells were treated with PVE for 24 h, under a serum-free condition. Next, MTT reagent (1 mg/ml) was added to each well and the cells were incubated for 3 h. The medium was then discarded and the cells solubilized with DMSO. The absorbance was measured at a wavelength of 570 nm using a spectrophotometer [24].

### 2.5. Transient Transfection and Luciferase Assay

The HaCaT cells were transfected with the 11*β*-HSD1 luciferase reporters (GeneCopoeia, Rockville, MD, USA) using SuperFect® Transfection Reagent (Qiagen, Hilden, Germany). After 24 h of incubation, the cells were incubated with PVE induced by cortisone for 24 h. Subsequently, the cells were harvested and lysed, and the supernatants were measured for their luciferase activity using a BioLux® Gaussia Luciferase Assay Kit (New England Biolabs, Ipswich, MA, USA), and an Infinite® 200 PRO luminometer (Tecan, AG, Männedorf, Switzerland).

### 2.6. Enzyme-Linked Immunosorbent Assay (ELISA)

Cortisol concentration was quantified in culture supernatants of HaCaT after treatment with PVE using a commercially available ELISA kit (Enzo Biochem, New York, NY, USA). Cell culture supernatants were gathered 24 h after treatment with PVE and assayed for cortisol. Procollagen type I was quantified in the culture medium of cocultured NHFs using a commercially available ELISA kit (Takara Bio Inc., Japan). The standard curve was linearized and regression analysis was performed. The cortisol concentration was determined using a standard curve.

### 2.7. Immunocytochemistry

Glucocorticoid receptor (GR) translocation was determined by immunocytochemistry. Typically, 2 × 10^2^ cells were plated in 96-well plates, cultured for 24 h, and treated with cortisone in the presence or absence of GAE for 40 min. The cells were fixed in 4% formaldehyde, permeabilized with 0.1% triton X-100, and incubated with a PE-conjugated antihuman GR (Novus Biologicals, USA) for 2 h. Subsequently, the cells were examined, using the IN Cell Analyzer 100 (GE Healthcare Lifesciences, Uppsala, Sweden).

### 2.8. Total RNA Extraction, cDNA Synthesis, and Quantitative PCR

Total RNA extraction was performed using RNeasy kit (Qiagen, Hilden, Germany); cDNA was synthesized using a PrimeScript 1st Strand cDNA synthesis kit (Takara Bio Inc., Japan), according to the manufacturer's instructions. The HSD11*β*1, COL1A1, and NR3C1 mRNA were measured by real-time quantitative PCR. The primer sequences were as follows: HSD11*β*1: forward 5′-CCAGAGATGCTCCAAGGAAAG-3′, reverse 5′-TGGTGCCAGCAATGTAGTGT-3′; NR3C1: forward 5′-TGTGCTGGAAGGAATGATTG-3′, reverse 5′-AGGGGTGAGTTGTGGTAACG-3′; COL1A1: forward 5′-GGACACAGAGGTTTCAGTGGT-3′, reverse 5′-CACCATCATTTCCACGAGCA-3′; GAPDH: forward 5′-TGCACCACCAACTGCTTAGC-3′, reverse 5′-GGCATGGACTGTGGTCATGAG-3′. All mRNA data were normalized to GAPDH expression.

### 2.9. *In Vitro* Coculture of Human Epidermal Keratinocytes and Fibroblasts

We used the Millicell® Cell Culture insert (Millipore, Billerica, MA, USA), according to the manufacturer's instructions. Briefly, human epidermal keratinocytes and human fibroblasts were cocultured in transwell system. For HEKn, 1 × 10^3^ cells were seeded in the upper chamber, with 8 *μ*m-pore filters and lower chambers were seeded with fibroblasts at 5 × 10^4^ cells for 24 h. PVE, 11*β*-HSD inhibitor, and cortisone were added to the upper wells. After 72 h of incubation, cell culture medium and NHFs were harvested, and ELISA and real-time qPCR were performed to measure the levels of collagen.

### 2.10. Statistical Analysis

Differences between the control and treatment group were analyzed by Student's t-test. A* P* < 0.05 was considered statistically significant.

## 3. Results and Discussion

### 3.1. Chemical Composition Analysis of PVE

**Figure 1:**
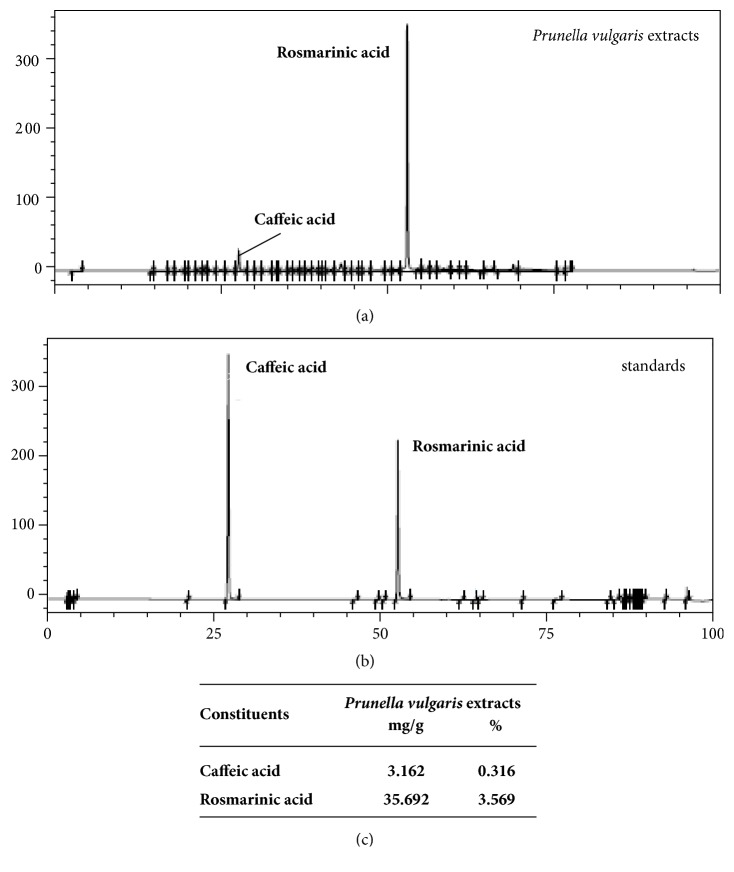
Phytochemical analysis of PVE (a, b). Composition of* Prunella vulgaris *extract (PVE). (c) Quantitative analysis of the phytochemical activity of PVE.

The PVE contained 0.3% caffeic acid and 3.5% rosmarinic acid. Chromatograms of typical extracts and the mixed standards are shown in Figures 1(a) and 1(b). The results of the quantitative analysis are summarized in Figure 1(c).

### 3.2. PVE Inhibits 11*β*-HSD1 Expression

**Figure 2:**
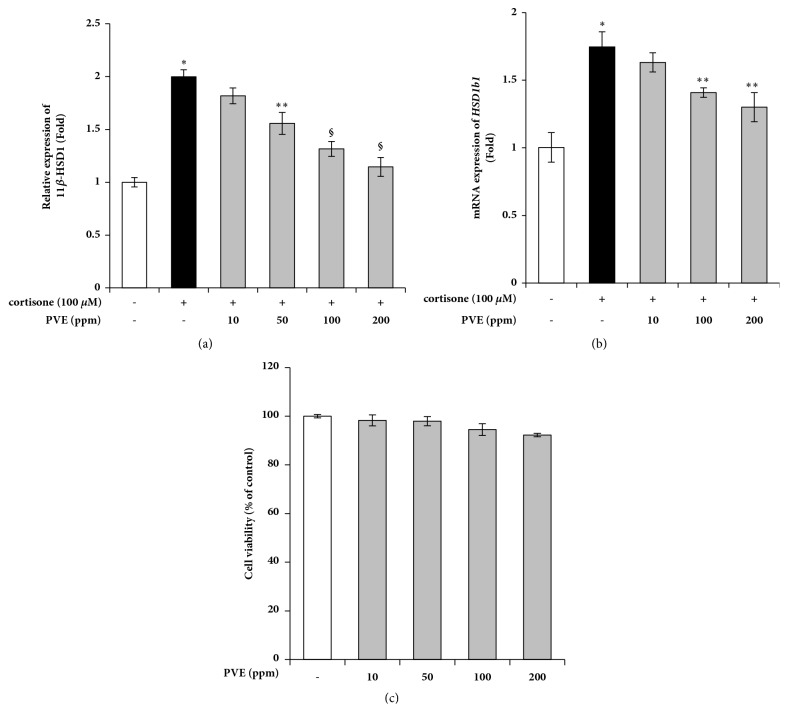
Effects of PVE on 11*β*-HSD1 expression in cortisone-induced HaCaT cells. (a) The 11*β*-HSD1 promoter-luciferase reporter vector was transfected into HaCaT cells and cultured for 24 h. The cells were pretreated with PVE for 1 h and then treated with cortisone. Luciferase activity was assessed against cortisone-treated control. (b) Cells were cultured in serum-free DMEM, in the presence of indicated concentrations of PVE, for 24 h. The level of 11*β*-HSD mRNA was assayed by quantitative real-time PCR. (c) Cell viability was performed by MTT assay. The results are mean ± standard deviation (SD) (n=3). *∗P *< 0.01* versus* cortisone-untreated control. *∗∗P *< 0.05* versus* cortisone-treated control. §*P *< 0.01* versus* cortisone-treated control.

To determine the inhibitory effects of PVE on 11*β*-HSD1, we investigated the effect of PVE on cortisone-induced 11*β*-HSD1 expression. A luciferase assay and real-time qPCR were performed to measure 11*β*-HSD1 expression. As shown in Figure 1(a), cortisone-induced activation of 11*β*-HSD1 promoter was significantly inhibited by PVE. In addition, PVE inhibited 11*β*-HSD1 mRNA levels induced by cortisone, in a concentration-dependent manner (Figure 1(b)). Cytotoxicity was not observed when the cells were incubated with 10 ~ 200 ppm of PVE 24 h (Figure 2(c)).

### 3.3. PVE Inhibits Cortisol Production in HaCaT Cells

**Figure 3:**
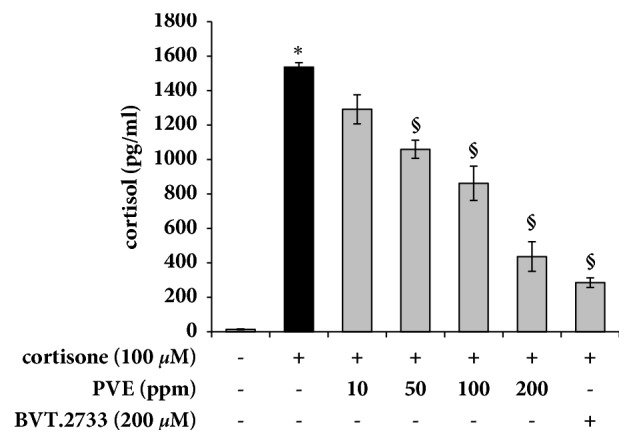
Effects of PVE on cortisol production in cortisone-induced HaCaT cells. The HaCaT cells were pretreated with PVE for 1 h before treatment with cortisone (100 *μ*M). The concentration of cortisol in the culture medium was measured by ELISA. The results are mean ± standard deviation (SD) (n = 3). *∗P* < 0.01* versus* cortisone-untreated control. §*P* < 0.01* versus* cortisone-treated control.

Keratinocytes are known to interconvert hormonally inert cortisone into active cortisol through the activation of the enzyme, 11*β*-HSD1 [6]. Thus, we investigated the effect of PVE on the production of cortisol in cortisone-induced HaCaT cells. The presence of PVE at a final concentration of 200 ppm significantly inhibited cortisol production (Figure 3). Comparable results were obtained with PVE, BVT2766 (a selective 11*β*-HSD1 inhibitor) on inhibition assay for cortisol production (Figure 3). These results suggest that the PVE-mediated inhibition of cortisol is associated with the inhibition of 11*β*-HSD1 activity.

### 3.4. PVE Inhibits Cortisone-Induced GR Translocation

**Figure 4:**
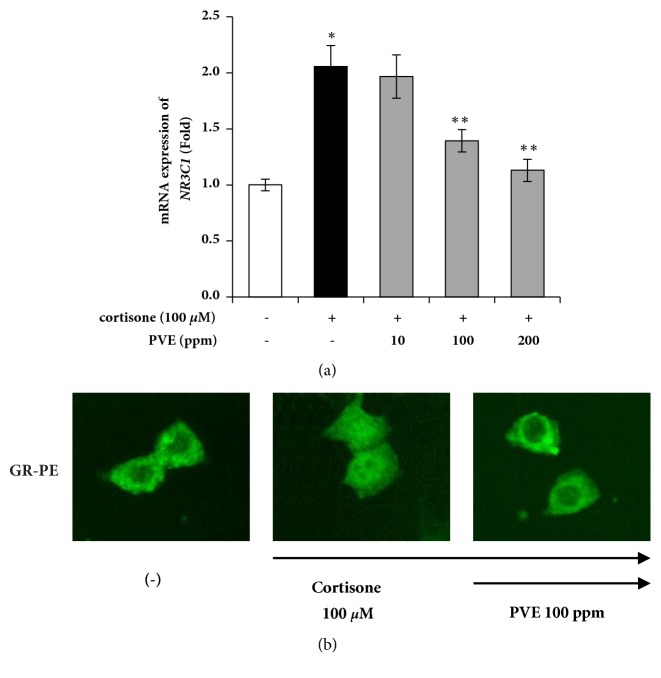
Effects of PVE on activation of GR in cortisone-treated HaCaT cells. (a) The HaCaT cells were cultured in serum-free DMEM, in the presence of an indicated concentration of PVE, for 24 h. NR3C1 mRNA levels were assayed by quantitative real-time PCR. (b) The HaCaT cells were treated with PVE for 1 h, before treatment with cortisone (100 *μ*M). After 40 min of stimulation, the cells were fixed, permeabilized, and incubated with PE-conjugated antihuman GR. GR-PE was analyzed using the IN Cell Analyzer 1000 instrument. The results are mean ± standard deviation (SD) (n = 3). *∗P* < 0.001* versus* cortisone-untreated control. *∗∗P* < 0.05* versus* cortisone-treated control.

The biological action of glucocorticoids is mediated by the glucocorticoid recep tor (GR), a member of the nuclear receptor superfamily of ligand-dependent transcription factors that are encoded by a single gene in human and mice called NR3C1 [25]. Once inside the nucleus, GR binds directly to glucocorticoid response element (GRE) and stimulates the transactivation of aging-related genes. We investigated whether PVE would regulate the expression and translocation of GR. The expression of the GR gene, NR3C1, was upregulated by cortisone, and its gene level was downregulated by PVE (Figure 4(a)). Subsequently, we performed immunocytochemistry for GR, to analyze GR translocation from cytosol to nuclei. The immunocytochemical studies revealed that externally added cortisone induces translocation of GR into the nucleus (Figure 4(b)). Simultaneous incubation with PVE blocked GR translocation, indicating inhibition of 11*β*-HSD1-dependent conversion of cortisone to cortisol (Figure 4(b)). These results suggest that GC-induced GR expression and nuclear translocation were inhibited by PVE (Figure 4).

### 3.5. PVE Prevents the Cortisone-Induced Collagen Decrease

**Figure 5:**
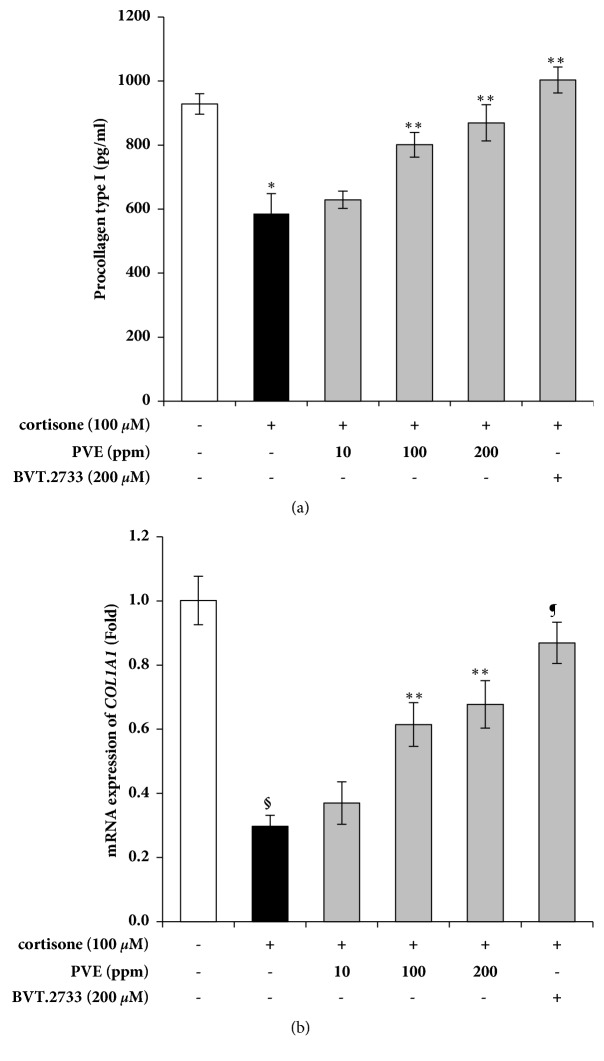
Effects of PVE on cortisol levels stimulated with cortisone in HEKn/NHFs coculture. The HEKn were seeded in upper chambers and NHFs were seeded in lower chambers of two-compartment transwell system for 24 h. Upper chambers were pretreated with PVE and 11*β*-HSD1 inhibitor, BVT.2733, and subsequently with cortisone. The HEKn were placed in the upper chamber with 8 *μ*m-pore filters. (a) Procollagen type I was measured using ELISA. (b) COL1A1 mRNA levels were assayed by quantitative real-time PCR. The results are mean ± standard deviation (SD) (n = 3). *∗P* < 0.05* versus* cortisone-untreated control, *∗∗P* < 0.05* versus* cortisone-treated control, §*P* < 0.01* versus* cortisone-untreated control, ¶*P* < 0.01* versus* cortisone-treated control.

Cortisol is known to reduce the cellular amounts of type I procollagen [26]. To investigate the effect of PVE on cortisol-induced inhibition of collagen synthesis, human epidermal keratinocytes (HEKn) were cocultured with human dermal fibroblasts (NHFs) using a transwell culture system. HEKn in the upper chamber were pretreated with PVE or BVT.2733, a selective 11*β*-HSD1 inhibitor, and then stimulated with cortisone. Collagen levels were significantly decreased upon exposure to 100 *μ*M of cortisone. The treatment with cortisone and either PVE or 11*β*-HSD1 inhibitor prevented the cortisone-induced collagen decrease at both mRNA and protein levels (Figures 5(a) and 5(b)). These results indicate that PVE-mediated inhibition of 11*β*-HSD1 can prevent the decrease in collagen levels upon exposure to cortisone.

## 4. Discussion

Systemic GCs produced by HPA axis have been studied in the laboratory and in clinical trials. Cortisol is one of the major endogenous GCs, which is released in response to a various physical stressor and psychological stress. Recent studies have focused on local cortisol production by their activating enzyme. The enzyme, 11*β*-HSD1 is known to catalyze the conversion of inert cortisone to active cortisol in skin cells. Skin is a target of key stress mediators and is a local source of inducing various immune and inflammation responses. However, augment innate and adaptive immune responses and chronic stress can have deleterious effects on the skin. Long-term topical GCs treatment leads to severe skin atrophy, including decreased epidermal thickness, flat dermal-epidermal junction, and disruption of the dermal fibrous network. Therefore, regulation of 11*β*-HSD1 is a target for maintaining skin homeostasis.

GCs have effects on dermis and epidermis that is connected to the basement membrane. Keratinocytes are the major constituents of epidermis attached to the basement membrane. The dermis is mainly composed of fibroblasts which are responsible for making the extracellular matrix and collagen as the main component of connective tissue. The basement membrane is composed of two layers, the basal lamina and the underlying layer of reticular connective tissue, which ensures the stability of connection and communication between these two compartments. Thus, the expression of 11*β*-HSD1 within the epidermis and the dermis represents specific mechanism in the regulation of GC activation.

In this study, we found that a selective inhibitor (in PVE) of 11*β*-HSD1 had a strong inhibitory activity on cortisol activation in keratinocytes. After diffusion into the cells, GCs bind to the intracellular GC receptor (GR), induce a structural change in the GR molecule, and translocate the activated GR to the nucleus. The translocated GC/GR complex binds to GRE and affects gene expression either positively or negatively. Therefore, antagonism of the GR has been used to minimize the detrimental effects caused by chronically elevated GC levels.

Water extract of PV is mainly composed of rosmarinic acid and caffeic acid (Figure 1). Rosmarinic acid, a major constituent, showed independent inhibitory activity of 11*β*-HSD1, whereas caffeic acid was not observed (supplementary Figure 1). Further active component identification and bioavailability studies will help reveal more about PV as a mediator of GC-associated skin disease.

The degradation of ECM proteins in fibroblasts results in loss of the fundamental mechanical properties of the skin structure. Type I collagen, a heterodimer composed of three *α*-chains encoded by COL1A1 and COL1A2 genes, is a major component of ECM [27]. Smad is essential for induction of type I collagen transcription and GR has a negative effect on Smad-activated gene transcription [28, 29]. GCs also decrease procollagen gene expression through GRE [30]. We have shown that PVE treatment of HEKn decreases 11*β*-HSD1 mRNA and cortisol levels stimulated by cortisone. These results concomitantly enhance collagen synthesis by regulating the GC mediated collagen degradation. Furthermore, studies on the regulation of various ECM proteins affected by GC may provide a more advanced approach to skin therapy. Therefore, 11*β*-HSD1 inhibitors may have beneficial applications in minimizing side effects associated with topical GC therapy such as skin atrophy [7].

## 5. Conclusions

We investigated the inhibitory effect of PVE on cortisol formation and found the inhibition of 11*β*-HSD1 as an underlying mechanism. Consistent with these results, cortisone-induced translocation of glucocorticoids receptor (GR) was also attenuated. These inhibitory effects of PVE showed potential in the regulation of collagen gene expression. Based on our results, it is proposed that PVE may be used for maintaining skin integrity by inhibiting 11*β*-HSD11 activity.

## Data Availability

The data that support the findings of this study are available from the corresponding author upon reasonable request.

## Supplementary Materials

Supplementary Materials Supplementary Figure 1: effects of caffeic acid, rosmarinic acid, and PVE on cortisol production in cortisone-induced HaCaT cells.
