# Supplementary material for: Inhibitory Effects of Prunella vulgaris L. Extract on 11β-HSD1 in Human Skin Cells
Source: Evid Based Complement Alternat Med. 2018 Oct 4;2018:1762478. doi: 10.1155/2018/1762478 (PMC6193348; doi:10.1155/2018/1762478)
Supplement: Supplementary Materials — Supplementary Figure 1: effects of caffeic acid, rosmarinic acid, and PVE on cortisol production in cortisone-induced HaCaT cells. [file 1762478.f1.pdf]

Supplementary Figure. 1

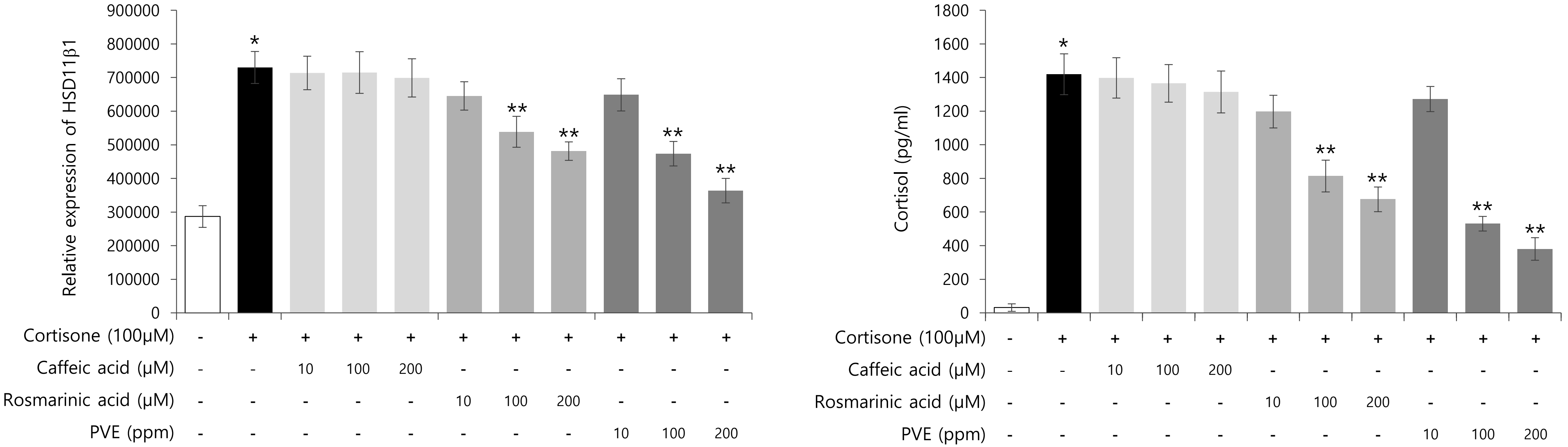

Supplementary figure 1. Effects of caffeic acid, rosmarinic acid, and PVE on cortisol production in cortisone-induced HaCaT cells. The HaCaT cells were pretreated with caffeic acid, rosmarinic acid, and PVE for 1 h before treatment with cortisone (100 μM). The concentration of cortisol in the culture medium was measured by ELISA. The results are mean ± standard deviation (SD) (n = 3). * $P < 0.01$ *vs*. cortisone untreated control, ** $P < 0.05$ *vs*. cortisone treated control.
